# A Computer Simulation Study of Anatomy Induced Drift of Spiral Waves in the Human Atrium

**DOI:** 10.1155/2015/731386

**Published:** 2015-10-26

**Authors:** Sanjay R. Kharche, Irina V. Biktasheva, Gunnar Seemann, Henggui Zhang, Vadim N. Biktashev

**Affiliations:** ^1^College of Engineering, Mathematics and Physical Sciences, University of Exeter, Exeter EX4 4QF, UK; ^2^Department of Computer Science, University of Liverpool, Liverpool LS9 3BX, UK; ^3^Institute for Biomedical Engineering, University of Karlsruhe (TH), 76131 Karlsruhe, Germany; ^4^Biological Physics Group, Department of Physics, University of Manchester, Manchester M13 9PL, UK

## Abstract

The interaction of spiral waves of excitation with atrial anatomy remains unclear. This simulation study isolates the role of atrial anatomical structures on spiral wave spontaneous drift in the human atrium. We implemented realistic and idealised 3D human atria models to investigate the functional impact of anatomical structures on the long-term (∼40 s) behaviour of spiral waves. The drift of a spiral wave was quantified by tracing its tip trajectory, which was correlated to atrial anatomical features. The interaction of spiral waves with the following idealised geometries was investigated: (a) a wedge-like structure with a continuously varying atrial wall thickness; (b) a ridge-like structure with a sudden change in atrial wall thickness; (c) multiple bridge-like structures consisting of a bridge connected to the atrial wall. Spiral waves drifted from thicker to thinner regions and along ridge-like structures. Breakthrough patterns caused by pectinate muscles (PM) bridges were also observed, albeit infrequently. Apparent anchoring close to PM-atrial wall junctions was observed. These observations were similar in both the realistic and the idealised models. We conclude that spatially altering atrial wall thickness is a significant cause of drift of spiral waves. PM bridges cause breakthrough patterns and induce transient anchoring of spiral waves.

## 1. Introduction

Atrial arrhythmias are complex phenomena involving a combination of electrophysiological [1, 2], structural [3], and anatomical [4, 5] factors. Persistent atrial arrhythmias are associated with ectopic beats [6], shortened action potentials [1, 7], and high rates of tissue excitation [8]. Due to the complex atrial electrophysiology and anatomy, complex rhythms arise as evidenced by multiple clinical and experimental studies [9, 10]. However, there is experimental evidence that spiral and scroll waves (SWs) play a prominent role in promoting atrial arrhythmia [11, 12] as reflected in recent experimental, computational, and theoretical studies [13–17].

SWs have been linked to subcellular electrophysiological ion channel remodelling [18–20] and the increasing stability of SWs studied extensively in multiple modelling studies in 2D tissue preparations [19, 20]. Fibre orientation structure in cardiac tissue has been shown to cause distortion, anchoring, and breakup of stable SWs according to Majumder et al. [21] and others. Other important mechanisms affecting SWs are breakthrough patterns associated with pectinate muscle (PM) bridges and are predominantly thought to be due to the complex arrangement of PMs on the atrial endocardial wall as observed in the sheep atria [17]. In extensive optical mapping studies, the Allessie group has shown atrial arrhythmia to be sustained by epicardial-endocardial dissociation leading the breakthrough patterns [22, 23]. The uneven cardiac anatomy also plays an important role in the dynamical behaviour of SWs [16, 17, 24–26]. The junctions of PM bridges and the atrial wall have been hypothesised to be anchoring locations for SWs by Yamazaki et al. [16]. Thus, the SWs may be drifting or pinned depending on heterogeneities in atrial anatomy and electrophysiology [16, 17, 26].

In contrast to studies exploring mechanisms of SW persistence, the drifting and pinning of SWs during an arrhythmia remain underexplored. Here, we explore the drift and pinning mechanisms of SWs induced by anatomy. The role of anatomy alone on atrial arrhythmia has not been extensively quantified to date, and therefore such an investigation is warranted. A general theory of perturbation induced SW drift in cardiac tissue has been discussed by Biktasheva et al. [27]. A nonuniform diffusive coupling has been shown to cause a spontaneous drift of SWs [28]. The genesis and drift of SWs in ischemic tissue involving gap junctional gradients and spatially heterogeneous cellular electrophysiology in cardiac tissue have been demonstrated by Biktashev et al. [29].

In the present study, the ideas of the theory of perturbation induced SW drifts are applied to the 3D atrial organ. The motivation for this computer modelling study is at the interface of theoretical mathematical developments and experimental atrial arrhythmia research. Based on idealisations of tissue homogeneity and simulation of stable SWs, we hypothesized that SWs are affected by anatomical perturbations giving rise to SW spontaneous drift. We interpreted the simulations in the realistic 3D model of the human atrium from the viewpoint of the previously developed mathematical theory [27, 30]. The theory states that a stationary scroll wave may spontaneously drift in response to small perturbations arising from electrophysiological, structural, or anatomical properties. Using both anatomically realistic 3D model of the human atrium and simplified 3D models, we demonstrate the role of atrial anatomy on the drift of SWs and identify individual causes of such drift.

## 2. Methods

Computational cardiology allows the systematic dissection of causal mechanisms of observed effects in the heart. As the aims of this study were to isolate the influence of anatomy on atrial SWs, the following assumptions were implemented:

- the electrophysiological model of ion current alterations was chosen that gave a stationary, nonmeandering SW;
- a constant diffusive intercellular coupling throughout the anatomy was assumed;
- the 3D atrium was assumed to be structurally isotropic without effects of fibre orientation.

These assumptions are justified by our focus on the specific effects of anatomical geometry on the evolution of SWs. Hence the complicating factors, such as meander of SWs, multiple wavelets, fibrosis, gap junctional heterogeneity, and fibre orientation, are excluded from consideration. All these factors are important, but they deserve their separate studies and hence are left beyond the scope of this paper.

### 2.1. Atrial Excitation Model

**Figure 1:**
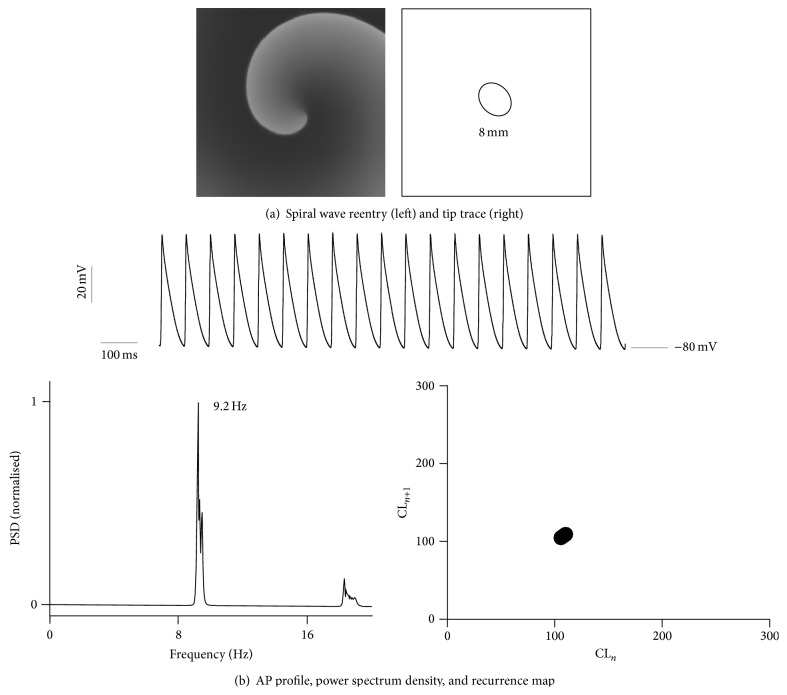
2D simulation of AF with stationary reentry. (a) Spiral wave reentry (left) with spiral wave tip trace (right). The diameter of the spiral wave core (8 mm) is indicated in the right hand side panel. (b) Analysis of representative AP profile (top panel) from the 2D simulation. PSD of the representative AP profile (bottom left) showing a peak frequency of around 9.2 Hz. Recurrence map (bottom right) of consecutive cycle length durations of AP profile indicates the monomorphic nature of the reentry.

The established human atrial action potential (AP) cell model by Courtemanche et al. [31] (CRN) was used in this study. The use of CRN model is justified while striking a balance in the use of a biophysically detailed cell model to simulate stable SWs and to maintain a reasonable cell model complexity. It is the single most widely used cell model [19, 32] shown to have similar AP properties to the more recent cell models [7] as far as this study is concerned. Electrophysiological changes to the CRN model were implemented as in previous studies [33, 34] to give stationary rotating reentry. This was achieved by reducing the maximum conductance of the L-type calcium current by 65%, along with a ninefold increase of slow delayed and the rapid delayed outward potassium currents. In addition, a simplified formulation of an acetylcholine-dependent potassium (*I*_KAch_) current was added according to the formulas:

(1) OAch=101+9.13/Ach0.47,CAch=0.052+0.451+eV+59.53/17.18,IKAch=Cm×OAch×CAch×V−Ek,

where *O*_Ach_ is the acetylcholine-dependent activation gating variable, *C*_Ach_ is the voltage dependent inactivation gating variable, [Ach] is the acetylcholine concentration taken to be 0.0035 *μ*mol/L in this study [33–35], *V* is the membrane potential, and *E*_*k*_ is the reversal potential associated with potassium currents. This defines an electrophysiological condition giving stationary spiral SWs in isotropic homogeneous sheets of atrial tissue (Figure 1).

The time stepping in all simulations was using Rush-Larsen technique for gating variables and forward Euler for all other variables, with a fixed time step of 0.01 ms. This time step was sufficiently small that further decrease did not affect simulation results.

### 2.2. Tissue Modeling

A monodomain formulation of cardiac tissue was used in this study. The formulation can be written as

(2) $$\begin{array}{c} C_{m}\frac{\partial V}{\partial t}=-I_{ion}+D\nabla^{2}V,\\ n\cdot \nabla V=0, \end{array}$$

where *C*_*m*_ is cell capacitance, *I*_ion_ is the total current, and *D* is the diffusive coupling that represents intercellular gap junctional coupling. The value of *D* was taken to be 0.07 mm^2^/ms to give a solitary wave conduction velocity of 0.4 mm/ms in the atria.

### 2.3. 3D Human Atrial Anatomical Model and Idealised Geometries

**Figure 2:**
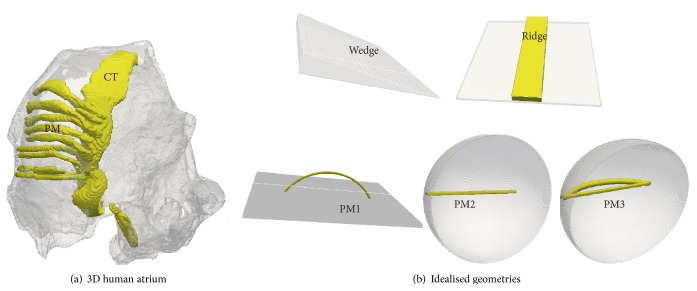
3D human atrium anatomical model and idealised geometries. In all geometries, the atrial wall is shown as translucent gray while the endocardial structures and their idealizations are shown as solid (yellow online). (a) 3D human atrial geometry showing uniform atrial tissue and anatomically variable tissue consisting of the conduction pathways, including CT and PMs. (b) The idealised geometries with wedge (variable wall thickness), ridge (constant thickness atrial wall with a ridge on it), a flat atrial wall with a curved PM bridge (PM1), a hemispherical atrial wall with a shorter pectinate muscle bridge (PM2), and a hemispherical atrial wall with a shorter PM bridge as well as a PM ridge that goes along the atrial wall between the junctions (PM3).

The human atrial anatomical model [25] used in this study is based on the Virtual Human Female data based on cryosection photographic images [36] and was assumed to be isotropic. To dissect the effects of anatomy, idealisations of the gross anatomical features were developed. All anatomical geometries considered in this study are illustrated in Figure 2. The gross anatomical features that were considered are (a) a varying wall thickness; (b) ridge-like formations made by PMs attached to the endocardial atrial wall or by the CT attached to the endocardial atrial wall; and (c) multiple bridge geometries to simulate PMs that are joined to the atrial walls only at their ends. The locations where the bridges join the atrial wall are referred to as junctions. Based on these observations, the following idealised anatomical models were developed: (a) wedge, where the atrial wall has a varying thickness; (b) ridge, where a central strip is thicker than the underlying atrial wall; and (c) multiple bridge geometries where a PM forms a bridge on the endocardial surface of the atrial wall. The first bridge geometry (PM1) was a flat-walled bridge forming the base of a curved PM. Such a geometry has been considered previously by Gray et al. [17], though they assigned a higher value of conduction velocity to propagations in the PM as well as considering a longer PM bridge. In the second bridge geometry, a curved atrial wall was taken with a PM bridge that formed a shorter connection between the two junctions of the atrial wall and the bridge (PM2). This geometry was then extended to include a ridge that went from one junction to the next but along the curved atrial wall (PM3).

The 3D anatomical model is formulated on a Cartesian grid enclosed in a box of dimensions of 77.55 mm (*x*) × 88.77 mm (*y*) × 98.34 mm (*z*) with an internode distance of 0.33 mm in each direction. The right atrial wall was estimated to have approximately 3 mm in thickness. The surface of the right atrium was estimated to have a size of approximately 25 mm × 25 mm. The maximum thickness of the PM and CT structures was estimated to be 2 mm. Therefore, in all the idealised geometry models, the atrial wall was taken to be 3 mm thick while the ridge and cylindrical bridge formations had a thickness (i.e., diameter) of 2 mm. The flat square surfaces representing the atrial wall in the wedge, ridge, and PM1 were taken to be 25 mm × 25 mm. The length of the curved PM bridge in PM1 geometry was taken to be 14 mm. In the PM2 and PM3 geometries, the curved atrial wall was formed of a hemisphere with diameter of 30 mm. The straight PM bridges in PM2 and PM3 were given a length of 8 mm corresponding to the length of the PM bridge estimated in the 3D atrial anatomical model.

In the wedge model, the thickness was gradually reduced from the maximum at the left end till zero thickness at the right end. An SW close to the thick end of the wedge was initiated and allowed to evolve. In the ridge and the bridge models, SWs were initiated at multiple locations far away, close to, or at the location of ridges, bridges, or bridge-atrial wall junctions. In all idealised geometry simulations, the space step was 0.33 mm. Test simulations performed at a more refined space step of 0.1 mm produced similar results.

The space discretization in all cases was on a Cartesian grid with constant space step and 7-point stencil for the Laplacian of (2), with the no-flux boundary conditions implemented using the methods of weights as in Clayton and Panfilov [37] and in ten Tusscher and Panfilov [38], both for the anatomically realistic geometry and for the idealized geometries.

### 2.4. Simulation of SWs, 3D Data Analysis, and Visualization

**Figure 3:**
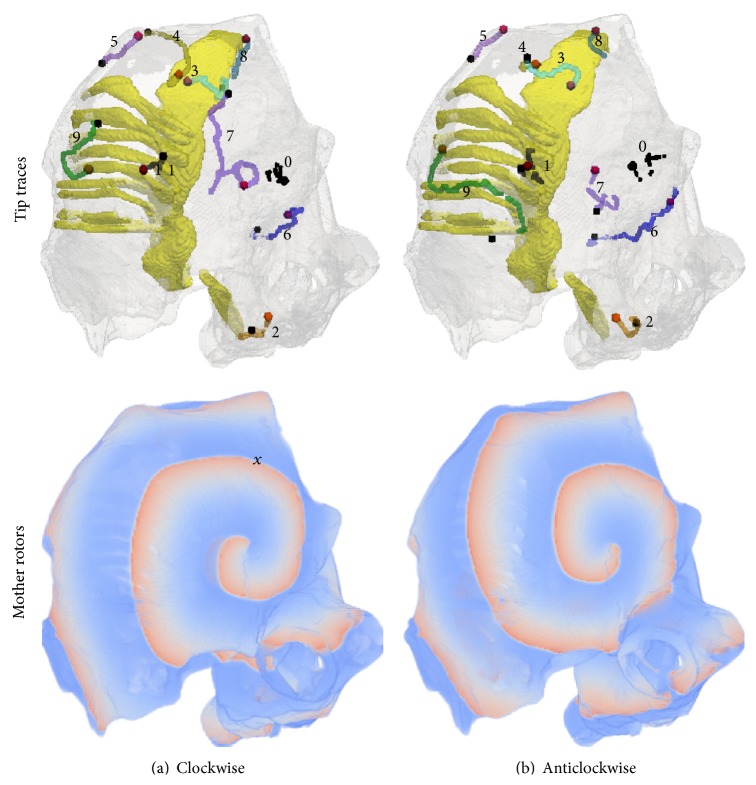
Summary of the anatomical model simulations. Column (a) shows data for clockwise and column (b) shows data for anticlockwise SW simulations. Top row shows nine selected tip traces of the spontaneously drifting scroll waves in different colours. On each trace, the red dot is the earliest point and the black dot is the latest point of the tip trace. The traces are also enumerated, and the numbers are referred to in the text. The initiation location of traces with the same numeric labelling is the same in the left and right panels. Bottom row shows typical snapshots of spiral waves seen on the surface nearest to the viewer. Left panel shows a clockwise rotating spiral while the right panel anticlockwise rotating spirals. The cross in the top right (left panel) designates the position of the registration electrode where a representative AP was recorded.

SWs were initiated at multiple locations in the right atrium or the idealised geometries by means of the phase distribution method [39]. Briefly, the phase distribution method of scroll wave initiation involves pacing of the cell model rapidly till steady APs as well as oscillations in all other state variables are obtained. The state variables over the span of one oscillation are then used to pace a 1D tissue to produce steady propagations in the strand. The state variables recorded from the middle of the strand during one steady propagating pulse are then used to distribute the phase of the electrical excitation to induce an SW according to a three-dimensional extension of a formula that gives Archimedean spiral shape of the lines of equal phases if applied on a 2D plane. Using such a protocol ensures the initiation of an SW at a prescribed location in the model. In the 3D anatomical model, a uniform grid of a total of 100 initiation sites was prescribed. At each location of this grid, rotors with either chirality, that is, clockwise and anticlockwise, were initiated in consecutive simulations. Chirality of a rotating wave is illustrated in Figure 3. Thus a total of 100 clockwise and 100 anticlockwise simulations in the 3D atrium were conducted. The SWs were allowed to evolve for a total duration of 40 s. The 3D voltage distribution was recorded at every 10 ms during the simulation. Further, a representative AP was also recorded at a site as shown in Figure 3. The evolution of the SW was quantified by computing the filament trace using the Montani et al. [40] version of the Marching Cube algorithm, implemented by Barkley et al. in EZ-SCROLL [41, 42], which is a broken-line approximation of the line of intersection of isosurfaces of the voltage variable,* V* = −50 mV, and the transient outward current inactivation gate variable *o*_*i*_ = 0.5. As the atrial wall is thin, the filaments at any time instant in our simulations were short and transmural. Therefore, only the epicardial ends of the filaments (hereafter termed as tips) were visualised for clarity. As the rotor core is circular, the averaged tip trace is shown in all results. The averaged tip trace was obtained by averaging the tip data over the course of one rotation of the SW. An SW was deemed to be drifting if its movement was more than 10 mm during a 40 s simulation, and if the movement of the tip was not interpretable in terms of continuous drift the simulation was not taken into account in this study. This allowed exclusion of transient initial conditions effects as well as overly complex drifting behaviour which is beyond the scope of the present study. Power density spectrum (PSD) of the registered AP profiles was computed to obtain the dominant pacing rates [18]. Recurrence maps were computed from the registered AP profiles to elucidate the form of the apparent arrhythmia, that is, monomorphic or polymorphic [43].

The visualisation was carried out using a combination of in-house software and VTK [44]. The 3D output files from Beatox cardiac simulation environment consisted of binary files which contained discretised data for three chosen dynamical variables from each location in the spatial model. These data were converted to binary VTK files and thereafter visualised using VTK pipelines. As the data were large (a total of 2.5 TB data were visualised), the visualisation was carried out noninteractively using the UK EPCC service as well as the University of Exeter HPC service. The tips were tracked noninteractively using the Marching Cubes algorithm as described above, and the resulting data converted to VTK files to allow superimposition of data.

### 2.5. The Simulation Environment, BeatBox

BeatBox is a multifunctional high performance computing cardiac simulation environment [45]. It incorporates biophysically detailed cell models and anatomical heart models to facilitate multiscale* in silico* cardiac simulation studies. The package implements explicit and implicit finite difference solvers. The file *I*/*O* functionality is also parallel in BeatBox. The output from BeatBox can be directly analysed by in-house developed algorithms for filament detection and tracking. It can simulate electrical propagation in idealised and realistic geometries. An easy-to-use inbuilt script interpreter allowed rapid construction of simulation projects without recourse to low level code manipulation. In the 3D human atrium simulations of this study, 256 processors yielded the 40 s of simulated electrical activity within 38 hours. The numerical efficiency of BeatBox is reflected in its highly scalable nature as shown previously [46]. This* in silico* study demonstrates the functionality of the BeatBox package in conducting large scale computational investigations that potentially impact clinical practice. The simulation environment is freely distributed under GNU license and can be obtained from http://empslocal.ex.ac.uk/people/staff/vnb262/software/BeatBox/index.html.

## 3. Results

### 3.1. Persistence of 2D Spiral Waves

Under the electrophysiologically remodelled conditions, the AP is drastically abbreviated with an AP duration (APD_90_) of 106 ms as compared to control conditions where APD_90_ is 302 ms [31]. Upon simulating spiral waves in a 2D homogeneous sheet of atrial tissue, the reentry is stable and persistent (Figure 1). The spiral wave tip trace shows no meander and is about 8 mm in diameter. Analysis of an AP recording from a representative location in the 2D model can be seen to have a dominant frequency of 9.2 Hz with minimum contributions from other pacing rates indicating a monomorphic atrial tachycardia. The recurrence map also shows a stable monomorphic period of 106 ms throughout the simulated interval of 40 s.

### 3.2. Observation of Spontaneous Drift in the Human Atrium

**Figure 4:**
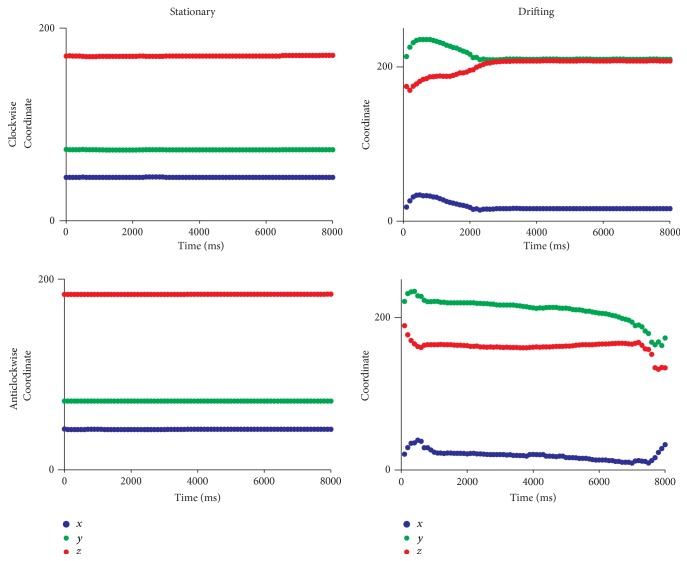
Plots of the scroll tip coordinates (*x*, *y*, and *z*) with respect to time for selected stationary (left column) and drifting (right column) trajectories. Clockwise spirals are shown in top row while anticlockwise spirals in bottom row. The stationary plots are from trace 0 as shown in Figure 3 in both clockwise (top) and anticlockwise (bottom) cases. The drifting plots are from trace 9 as shown in Figure 3 in both clockwise (top) and anticlockwise (bottom) cases. In the drifting cases, data for the first 8 s is shown after which the SWs became stationary.

**Figure 5:**
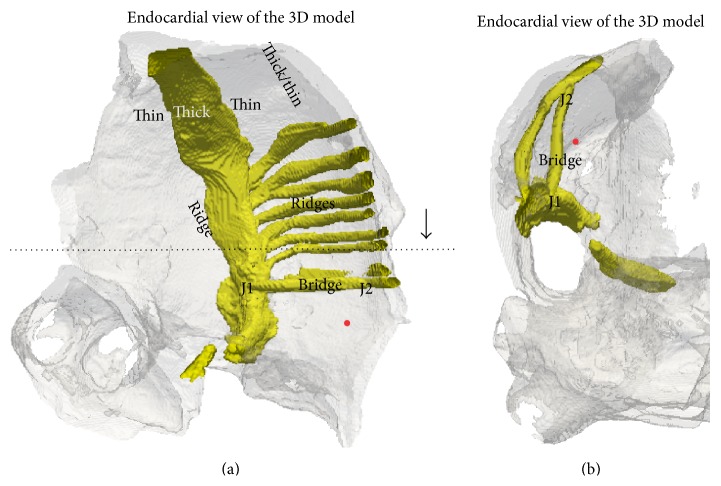
Cross-sectional endocardial views of the atrial geometry. (a) Overall anatomical structures considered in this study that give varying wall thickness, ridges, or bridge formations. The dotted line shows where a further cross section was taken and the arrow indicates the view of panel (b). A red dot is placed which indicates the orientation of panel (b) with respect to this panel. (b) The PM bridge in the 3D anatomical model which joins the atrial wall only at junctions J1 and J2.

Selected tip trajectories of SWs in the 3D atrium simulations are shown in Figure 3. In parts of the atrium devoid of significant anatomical features, the SWs were stationary (Figure 3, tip trace 0 of both chiralities). A closer examination of the local atrial anatomy revealed that there are no appreciable alterations in atrial wall thickness and those regions were also devoid of ridge-like and bridge-like structures. Stationary SWs without drift, apart from a minor initial transient movement within a small area, were observed also in regions of large anatomical heterogeneity (Figure 3, tip trace 1, both chiralities). In several simulations, a significant drift of the SWs was observed (Figure 3, tip traces 2 to 9 of both chiralities, except for trace 4 in the anticlockwise simulation which has no drift although it is close to a ridge). Typical features of such drift were (a) a rapid initial transient; (b) a continuing drift along anatomical structures; (c) eventual apparent anchoring of the SW. When the chirality of the SWs was reversed from clockwise to anticlockwise, the behaviour of SWs starting at the same location was changed; see Figures 3 and 4. In Figure 4, the drift is illustrated by plotting the *x*, *y*, and *z* coordinates of the tip with respect to time. In tip traces 3, 4, and 5 of both chiralities in Figure 3, a drift of the rotors from thicker regions to the closest thinner regions was observed. In case of tips 3 and 4 (both chiralities), the drift was from the thicker CT region into the thinner adjoining atrial wall. In case of tip trace 5, the drift was from the thicker right atrial appendage region to relatively thinner regions. The online supplementary videos show the time course of the drift of trajectory 5 in the clockwise (Online Supplement, S1 available at http://dx.doi.org/10.1155/2015/731386 ) and anticlockwise (Online Supplement, S2) cases. Apart from a varying wall thickness, ridge-like formations were also found to influence the evolution of the SW tip trajectories in their own way. In case of tip traces 6, 7, and 8, the drift proceeded along such ridges. In case of tip trace 6, the drift was along a ridge formed due to a sudden change in atrial wall thickness from right atrium to left atrium. In case of tip traces 7 and 8, the drift was along the CT ridge with the atrial wall. Tip trajectory 9 is near not only ridge-like formations made by PMs that are connected with the atrial wall along all of their lengths but also a bridge-like formation made by one of the PMs that is connected to the atrial wall only at its ends. This is illustrated in the endocardial surface view of the right atrial wall region as shown in Figure 5. In previous experiments and in simulations, breakthrough patterns were observed to emerge from the junctions of this bridge-like PM with the atrial wall, and the SW drifted towards the junctions [17, 18, 47–49]. Indeed, as trajectory 9 of the anticlockwise case shows, there is a definite drift of the SWs towards the junction of the PM. The online supplementary videos show the time course of the drift of trajectory 9 in the clockwise (Online Supplement, S3) and anticlockwise (Online Supplement, S4) cases. Having identified some specific behaviour of SW that is influenced by atrial anatomy, we conducted idealised geometry simulations.

### 3.3. Exploring the Mechanism for Spontaneous Drift in the Atrium Using Idealised Geometries

Based on the observations of SW drift in 3D atrial geometry and theoretical knowledge of spiral and SW drift available from previous literature, we identified the following possible “elementary” mechanisms responsible for the observed drift:

- a “wedge” that is a gradual alteration of atrial wall thickness, which can cause the SWs to drift at a certain angle with respect to the thickness gradient [30];
- a “ridge” that is a sudden change in atrial wall thickness which can cause the SWs to migrate along the ridge [27];
- a “bridge” made by a PM which may cause breakthrough patterns that affect the rotors. We explored three variants of the bridge geometry to elucidate the role of the PM bridge.

We tested the feasibility of these mechanisms by reproducing them in idealized geometries where only one feature was present in each simulation to explain the SW behaviour as seen in our 3D simulations with the anatomically realistic atrial model. A composite bridge-ridge geometry was also used to explore a more realistic structure of the bridge formation as observed in the 3D model (Figure 5(b)). Due to the simplicity and symmetry of the idealised geometries, SW simulations with only one chirality were conducted.

#### 3.3.1. Wedge

**Figure 6:**
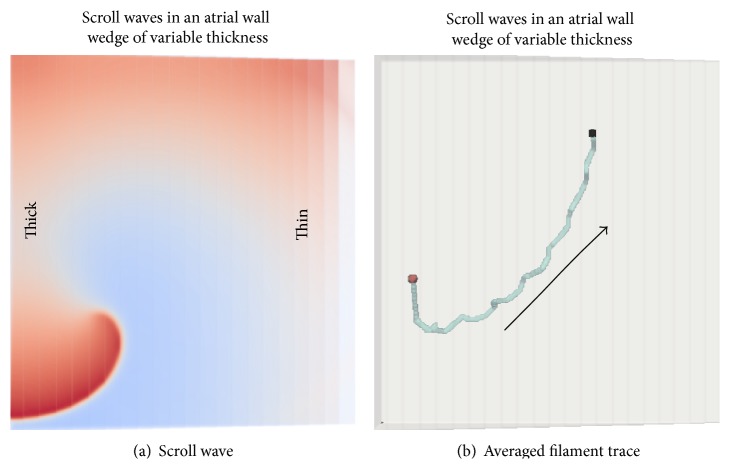
Wedge simulation. (a) A snapshot of a surface spiral wave during an early stage of the simulation. (b) Trajectory of the tip. The direction of the drift is from thick to thin part of the wedge as indicated by the arrow.

In the anatomically realistic model, the drift of SWs from thicker to thinner regions of the atrial wall was observed as shown by tip traces numbers 3 and 5 (both chiralities) in Figure 3. The idealised wedge simulation is illustrated in Figure 6. An online supplementary video shows the time course of the drift in a wedge (Online Supplement, S5). The SW that was initiated in one corner of the thick end of the wedge spontaneously drifted to the thin end. This is consistent with the drift of scrolls in 3D atrial simulations through smooth parts of the atrial walls towards thinner loci and is an evidence that the SW filament tension in this model is positive, so that the filament length tends to get shorter as the SW drifts. The theoretical basis for the filament tension concept stems from Biktashev et al. [30].

#### 3.3.2. Ridge

**Figure 7:**
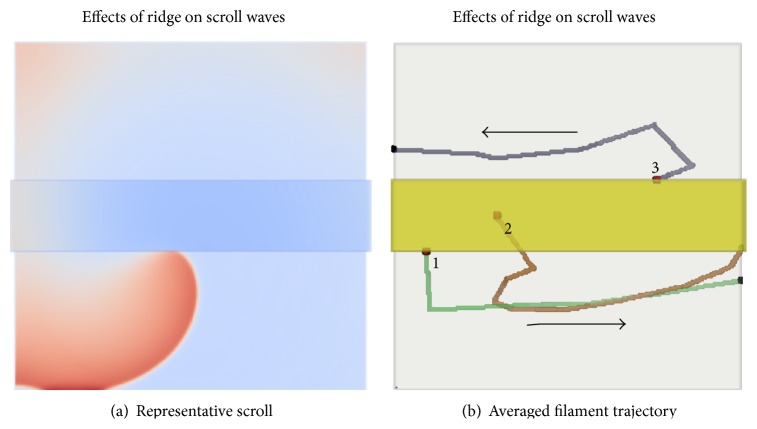
Ridge simulation. (a) A snapshot from the simulation where the SW was initiated close to the ridge. (b) Tip traces from 3 representative simulations where the scroll wave was initiated on the ridge (traces 1 and 3) or in the middle of the ridge (trace 2).

In the anatomically realistic model, there are several ridge-like formations, that is, sudden increases of atrial wall thickness protruding on the endocardial side. In our anatomically realistic simulations, the tips numbers 6, 7, 8, and 9 drift along ridges. SWs were simulated in an idealised 3D ridge model as shown in Figure 7. When SWs were initiated sufficiently far away from the ridge, there was no drift. However, when SWs were initiated close to the ridge, the SWs first underwent a transient motion into the thinner region of the model. Thereafter, the SWs drifted rapidly along the ridge, predominantly through the thin regions, until eventually reaching the boundary of the model. It should be noted that the movement along the ridge was much faster than in the wedge model case. Depending on which side of the ridge the SW was initiated, it was observed to travel in opposite directions. This was verified in the idealised geometry and is consistent with the symmetry of the idealized geometry with respect to rotation by 180 degrees. Also, it follows from the *x* → −*x* (where *x* is the horizontal coordinate in Figure 7(b)) symmetry of the model that the opposite direction of the drift would be observed for an SW on the same side of the ridge but of opposite chirality. This can be correlated to trajectory 9 in Figure 3, where the clockwise and anticlockwise SWs drift in opposite directions to each other. Online supplementary videos show the time course of the drift in the ridge when initiated at various locations (Online Supplements, S6, S7, and S8). Some theoretical insight into this phenomenon can be obtained by comparison with simulations illustrated in Figure 4(g) in [27]. In that simulation, an attachment of a spiral wave to a stepwise parametric inhomogeneity and subsequent stable drift along it was described and explained theoretically. This is a phenomenon separate from filament tension and is related to nonmonotonic behaviour of the so-called response functions of spiral waves. With that behaviour of response functions, a spiral wave can be attracted to or repelled from the same inhomogeneity depending on the distance to it and hence existence of stable distances at which repulsion changes to attraction. Dynamics of excitation waves in thin layers with variable thickness can be described asymptotically by a two-dimensional model, in which thickness variation is a perturbation (this theory is deferred to another forthcoming publication). This makes our present case similar to the parametric inhomogeneity considered in [27]. So, although we have not calculated the response functions for the current model, the observed drift along a ridge is an empirical evidence of their nonmonotonic behaviour.

#### 3.3.3. Bridges

In the anatomically realistic simulations, the PMs are finger-like structures, typically running along the endocardial side of the right atrial wall and making ridge formations. However, as shown in Figure 5, there are also PMs which form junctions with the atrial walls with the muscle body being unconnected to the atrial wall. We shall call the junctions of such a bridge J1 and J2. We have considered the following idealized bridge geometries.

**Figure 8:**
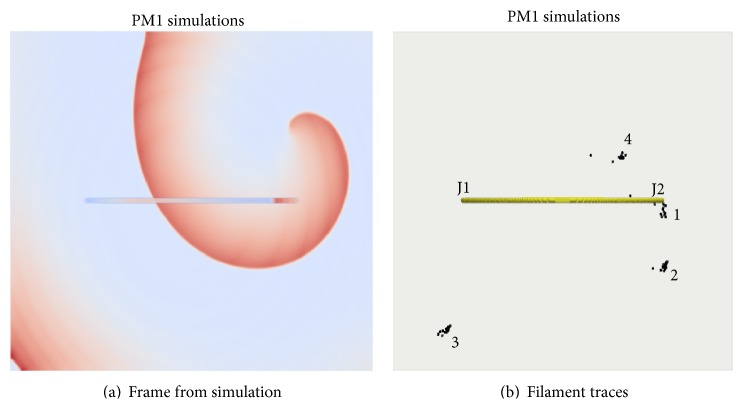
Flat wall bridge simulation. (a) Representative snapshot. (b) Tip traces from simulations where scroll waves were initiated close to the bridge (trace 1) or far away from the bridge (trace 3) or at intermediate locations (2 and 4).

**Figure 9:**
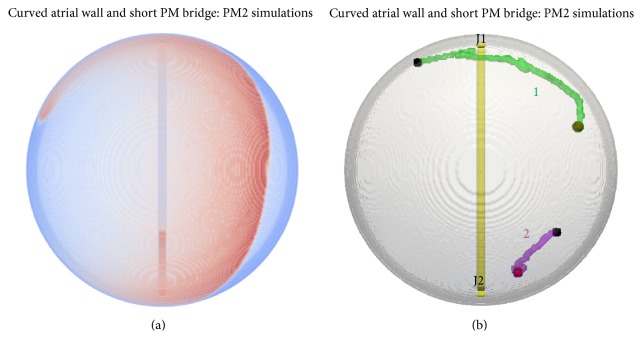
Curved wall bridge simulations. (a) Representative snapshot. The reentry looks distorted as it is on the steep part of the hemispherical wall, at a large angle to the presented view. (b) Two tip traces from simulations where scroll waves were initiated on either side of the bridge junctions. J1 and J2 indicate junctions of the PM bridge with the atrial wall on the endocardial side of the model.

**Figure 10:**
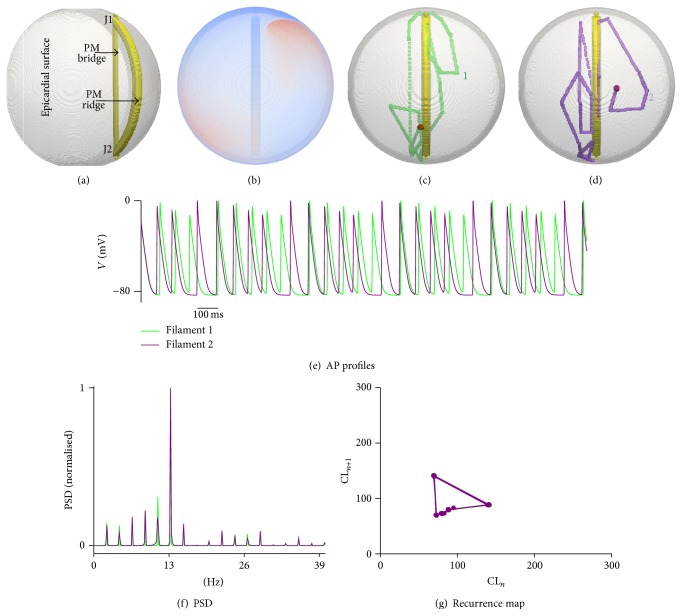
Curved wall bridge with ridge simulations. (a) Epicardial view of the idealised model showing the bridge and the ridge in solid yellow, while the hemispherical atrial wall is shown as translucent. The bridge and ridge are below the atrial wall (i.e., on the endocardial side) in the presented view. (b) Representative snapshot from SW simulation. (c) and (d) Two partial tip traces where the initiation locations are shown by red dots and the tip trajectories are shown by translucent green (trajectory 1, left panel) and translucent purple (trajectory 2, right panel). The trajectories continue to drift around the structure and do not have an end location. (e) AP profiles recorded from the two simulations in panel (c), showing a periodic increase and decrease of AP amplitude. (f) PSD showing the multiple dominant frequency components in the AP profiles of panel (d). (g) Recurrence map of AP profiles of panel (d) showing multiple cycle lengths of localised pacing due to the complex drifting pattern of trajectories 1 and 2.

(1) In a configuration such as flat atrial wall with a semicircular bridge (PM1 geometry in Figure 2(b)), the distance between J1 and J2 along the atrial wall is noticeably shorter than along the bridge. Therefore, regardless of where the SW was initiated, activation wave fronts always entered through both J1 and J2 in opposite directions into the bridge. The bridge, unsurprisingly, had no effect on the SW and it remained stationary. Tip traces from a few simulations that illustrate the stationary nature of the SWs when initiation sites were close to or far away from J2 are illustrated in Figure 8. An online supplementary video illustrates the time course of the drift in such a flat-walled bridge (Online Supplement, S9).

(2) In the actual 3D anatomical model, the PM bridge is short and the atrial wall is curved. We therefore simulated SWs in the model with curved wall (PM2 geometry in Figure 2(b)). Even though the PM bridge length between J1 and J2 is shorter than the distance along the wall, infrequent wave-break patterns were observed during the 40 s simulation. Irrespective of where the SW was initiated, the SW arm gave rise to colliding propagations to go through the PM bridge. The location of collision progressively moved gradually from one junction to the other. This affected the SW and caused a slow but definite drift closer to the junction where the collision location was moving towards. Eventually in case of trajectory 1 (Figure 9(b)), the SW core was almost coincidental with J2 and breakthrough patterns also occurred at the same junction. The direction of propagations through the bridge was observed to be reversed when the SW reached J2. The breakthrough patterns emerging at J2 then promoted the SW to move away from that junction. The movement of the tip was faster when the SW was at J2 and when the wave-break patterns started to emerge at J2. Depending on where the SW was initiated, it eventually settled at a certain distance (as shown in Figure 9) from the junctions. Such equilibrium was achieved due to the SW arm allowing propagations through J1 and J2 which collided exactly in the middle and therefore did not offer any perturbations (small or large) to cause further drift of the rotor. An online supplementary video shows the time course of the drift in this geometry (Online Supplement, S10).

(3) In the final idealised bridge geometry (PM3 geometry in Figure 2(b)), one PM formed a bridge between J1 and J2, while another formed a ridge along the atrial walls. This is shown in Figure 10. An online supplementary video further illustrates the time course of the drift in the PM3 geometry (Online Supplement, S11). Irrespective of where the SW was initiated, it rapidly drifted towards and then along the ridge forming PM. Such a swift drift was accompanied by an increased breakthrough pattern occurrence from J1 and J2 due to accompanying propagations through the PM bridge. In such a situation, the SW meandered rapidly between the two junctions along the ridge, while the bridge assisted in causing breakthrough patterns. The SW was also observed to spend prolonged durations at J1 and J2 (see Online Supplement Video S11). This complex propagation pattern is reflected in the representative recorded AP profiles (Figure 10(e)) where the AP amplitude was seen to periodically increase and reduce. In a marked difference to all other simulations presented in this study, several peaks were observed in the PSD of the AP profiles as shown in Figure 10(f) indicating the polymorphic nature of the excitation pattern. This was further confirmed in the recurrence map where consecutive cycle lengths had a periodic long/short variation. Such excitation patterns are known to be associated with Torsade de Pointes (TdP) where a periodically varying ECG is observed [50, 51].

Existence of a bridge is not a small perturbation in any sense, and therefore the asymptotic theory [27, 30] cannot explain its effect. Previous numerical studies have explored some of the effects of bridges [16, 17]. They however combined the effects of anatomy and inhomogeneity of diffusion coefficient (i.e., conduction velocity was considered to be higher in the bridge) or considered truncated PM structures.

## 4. Conclusions and Discussion

In this paper, we have studied the spontaneous evolution of a persistent single SW in the human atrium. The behaviour of SWs was simulated and quantified in terms of the SW drift and apparent anchoring. Further, the hypothesised mechanisms that lead to such spontaneous drift have been examined in idealised geometries which helped to separate and highlight the individual causes of the drift. The hypothesis that a variable wall thickness leads to drift of an SW was verified in this study. Another important feature of the atria is ridge-like formations in the PMs and the CT which form parts of the main conduction pathways in the organ. Even as the fibre orientation heterogeneity in the CT was not considered, we have seen that SWs tend to migrate along these ridges (Figure 3). This behaviour of the SWs was confirmed in the idealised ridge simulations. Thus one of the causes of SW migration is possibly due to sudden change in atrial wall thickness, as seen at the ridges formed by CT and atrial wall, and those of the PM and atrial wall (Figure 5).

Apart from the CT and PMs forming ridges, the PMs also form an intricate network of conduction pathways with bridge-like structures in the atrium. A typical effect of such bridges in the atria was seen to be an emergence of breakthrough patterns. These patterns interact with the SW causing it to migrate. Gray et al. [17] have demonstrated this in numerical simulations. In those simulations, the PM had a higher conductivity, thereby supporting the emergence of breakthrough patterns through the PM bridge and the SW location was selected close to a PM-atrial wall junction so as to allow a unidirectional propagation in the bridge. Such precise initial location is unlikely, and our present results indicate that breakthrough patterns can be observed even as SWs are initiated at not such specific locations. Further, no assumption of higher conductivity in the PM bridge was required to demonstrate that a bridge is capable of supporting breakthrough patterns. If we were to include fibre orientation induced rapid conduction in the PM bridge, it is possible that we would produce breakthrough patterns with an increased likelihood. In another study, Wu et al. [49] have seen erratic propagations in canine atria, evolving into high pacing rate excitations, thus indicating anchoring to PM junctions, where PM bridges formed parts of reentry circuits. They demonstrated, using experimental data as well as idealised geometry based numerical data, that the base of the PM can be an anchoring site and that PM bridge can be a substrate for reentry. Wu et al. [49] as well as Yamazaki et al. [16] argue that the junction of PM and atrial wall can be an anchoring site due to the varying thickness of atrial wall in its vicinity. The PM itself is an elongated structure capable of conducting excitations by pathways alternative to the atrial wall. Thus, if a PM forms a bridge, then it most likely causes breakthrough patterns, thereby affecting an SW and not always causing anchoring. In our simulations, in both the anatomical and the idealised geometries, we have considered this aspect and showed that the junctions of PM bridges with the atrial wall can be apparent sites of transient anchoring as well as breakthrough patterns which cause drift of SWs in the atrial wall. Previous studies [16, 17, 49] did not take into account the shorter length of the PM bridge as compared to the curved atrial wall or considered a PM bridge junction in isolation. In this study, we isolated and explored the role of the uneven atrial anatomy in the context of stable SWs. As the PM3 simulations in this study show, an increasing amount of complexity in the anatomy leads to increasingly complex excitation patterns. Apart from the PM3 simulations, all SW simulations are essentially monomorphic characterised by a single dominant frequency of approximately 10 Hz and a highly localised recurrence map. In case of the PM3 simulations, a simple addition of a PM ridge in addition to a PM bridge gave TdP type of excitation patterns. The ridge also assisted in temporary anchoring of the SW to the junctions, although it was not persistent. We see that relatively simple geometries (i.e., PM3) can to certain extent explain the movement of SWs as well as providing a plausible mechanism for apparent anchoring of SWs. The PM3 simulation results may provide evidence for anatomy based evolution of the SWs, which may manifest as large/small amplitude modulation of the ECG signal [52, 53].

### 4.1. Discussion

Pandit and Jalife [32] have recently reviewed the mathematical modelling as well as experimental literature on SWs and rotors. Reentry has been shown to be generated and sustained by means of focal activity in AF patients [54]. Our* in silico* study allowed the elucidation of the effects of atrial anatomy on such atrial reentrant SWs, albeit without the focal activity. Simulations presented in this study show that SWs tend to spontaneously drift due to anatomical features. The SW becomes localised or anchors to locations where it does not experience further perturbations or when multiple perturbations cancel out each other's effects. Unlike previous studies, in our simulations a PM bridge on its own did not form an SW anchoring location. It does however provide a conduction pathway that may give rise to complex wave patterns. The understanding provided by this study has several clinical implications. Firstly, it will assist in further developing efficient, low-amplitude defibrillation schemes [33, 55–57]. Secondly, clinical MRI based patient specific evaluation of gross anatomy can be used in predicting the eventual locations of the arrhythmic substrate, thereby personalising therapy. Results from this study can contribute to prediction of SWs in the proximity of such surgical remnants of anatomical deformities. Indeed, the presented computational framework can be used to predict the evolution of atrial arrhythmia given a reasonable patient specific atrial anatomy alone.

### 4.2. Limitations and Future Work

A single case of AF induced electrophysiological changes was considered in this study that gave stationary rotors using the CRN human atrial cell model. The stationary rotors were simulated using three electrophysiological alterations: (1) the *I*_KAch_ repolarising current was included, with an acetylcholine concentration of 0.0035 *μ*mol/L; (2) a 65% reduction of the L-type Ca^2+^ depolarising current was implemented; and (3) a 9-fold increase of the major repolarising potassium currents was implemented. This leads to a significant reduction of APD which may be considered nonphysiological; however the same or similar alterations of the CRN human atrial cell model were used in previous modelling studies for the purposes of theoretical analysis [33–35]. In our present case, these alterations allowed us to minimize any movement of SW purely due to the underlying electrophysiology and thus to isolate the effects of the anatomy on SW dynamics. Unlike some previous studies [58–60], the SWs in the atria in our model were largely stable and persistent without giving rise to wave breakup. The persistence of the SW was observed even as the arm of the rotor gave rise to complex electrical propagations in the uneven geometry. This is probably related to the choice of the CRN model and its specific modifications. In future studies, meandering SWs based on experimental electrophysiological data [7, 61–63] will improve our understanding of the behaviour of rotors in the atria. Conclusions drawn in this study rely on one anatomical model of the human atria which may be considered a limitation. Although it is known that human atrial anatomy is highly irregular, MRI data from AF patients and multiple anatomies may improve our evaluation of SW behaviour. Recent studies indicate that fibre orientation heterogeneity may be a key factor [64, 65], potentially superseding the uneven anatomy, in the genesis of complex dynamics during episodes of AF. In future studies, the role of such anisotropy will be taken into account, which will make the simulation results more realistic. In addition to AF induced ionic remodelling and the existence of fibre orientation, AF patients are known to have atrial diffusion inhomogeneity as well as advanced fibrosis [66]. SW interaction with such inhomogeneity is expected to give rise to SW breakup and erratic propagations and requires further investigation.

## Supplementary Material

The supplementary material consists of 11 animations. The animations illustrate the movement of of scroll waves in realistic or idealised geometries. Each animation shows 60 s of simulated electrical activity in the realistic atria (animations S1 to S4), or in idealised geometries (animations S5 to S11). The spiral wave filament trajectory on the surface of each anatomical model is shown as a solid black line. The electrical wave's propagating front is shown as red whereas resting atrial tissue is shown as blue. Each frame in the animation was stroboscopically recorded as described in the main manuscript.

## Abbreviations

AP: Action potential
APD_90_: AP duration at 90% repolarisation
AF: Atrial fibrillation
CL_*n*_: *n*th cycle length of an AP profile
CT: Crista terminalis
CRN: Courtemanche et al. (1998) human atrial cell model
J1, J2: Junctions of PM with atrial wall
SW: Spiral or scroll wave
PM: Pectinate muscle
PM1, PM2, PM3: Idealised geometries consisting of atrial wall, pectinate muscle bridges, and ridges
PSD: Power spectrum density
TdP: Torsade de Pointes.
